# Genetic diversity and the application of runs of homozygosity-based methods for inbreeding estimation in German White-headed Mutton sheep

**DOI:** 10.1371/journal.pone.0250608

**Published:** 2021-05-06

**Authors:** Sowah Addo, Stefanie Klingel, Georg Thaller, Dirk Hinrichs

**Affiliations:** 1 Institute of Animal Breeding and Husbandry, Kiel University, Kiel, Germany; 2 Department of Animal Breeding, University of Kassel, Witzenhausen, Germany; 3 Arche Warder, Center for Rare and Endangered Domestic Animals, Warder, Germany; University of Bologna, ITALY

## Abstract

The German White-headed Mutton (GWM) sheep is a monitoring population believed to have been improved through crosses with other breeds, e.g., Texel (TXL) and French Berrichone du Cher (BDC). The primary aim of the study was to analyse genetic diversity and breed composition of GWM sheep. Furthermore, different measures of computing inbreeding from the runs of homozygosity (ROH) were investigated. Data for GWM consisted of pedigree information on 19,000 animals and 40,753 quality filtered SNPs on 46 individuals. Additionally, publicly available genotype data on 209 individuals belonging to nine sheep breeds were included in the analysis. Due to evenness of SNPs spacing and proportionality of the number of SNPs in each autosome to autosome length, a high correlation (*r*_*p*_ = 0.99) was found between genomic inbreeding coefficients computed based on the length of ROH (F_ROH_L_) and those computed relative to the number of SNPs in ROH (F_ROH_N_). Total inbreeding was partitioned into values for individual chromosomes revealing the highest levels of inbreeding on chromosomes 1, 2 and 3. Correlations between the ROH-based inbreeding measures and pedigree inbreeding reached 0.82. The observed heterozygosity estimate in GWM was high (0.39), however, the breed suffered low level of effective population size (~50) from a genomic viewpoint. Moreover, effective number of founders (186), and effective number of ancestors (144) implied disequilibrium of founder contribution and a genetic bottleneck in the breed. Multidimensional scaling and network visualisation analyses revealed close connectedness of GWM to BDC and German Texel (GTX). A model-based admixture analysis consistently indicated the flow of genes from other breeds, particularly BDC to GWM. Our analyses highlight the mixed genetic background of GWM sheep and furthermore, suggest a close monitoring of the breed to consolidate its genetic diversity while averting further reduction in the effective population size.

## Introduction

Domestic sheep (*Ovis aries*) are important livestock species of most agricultural-based economies. They serve a variety of functions including the provision of products such as meat, milk, wool, skin and horn; by-products in the form of manure for fertilisation and dung for fuel or biogas production; and other benefits including landscape maintenance and dike protection, and a source of income and sociocultural prestige [1, 2]. The demand and supply of sheep and specific sheep products can have a profound influence on the breeding objective of sheep industry. For instance, in Germany, sheep breeders in the 1870s had to abandon the breeding of wool sheep in favour of mutton or dual-purpose sheep production [3]. Change of the breeding focus stem from a surge in the world wool production that made the local market unprofitable. Additionally, a sharp rise in human population growth necessitated changes in policies to allow for an increase in food production in the country.

Around the sixties and seventies of the 19^th^ century, the German White-headed mutton sheep (GWM) was developed in Northern Germany, originally, from a cross between the local Marsh sheep (Wilstermarschschaf and Butjadingen) and English breeds such as Cotswold, Leicester, Hampshire and Oxfordshire [3, 4]. The breed was formally recognised in 1885 [4] and later, further developments took the form of crosses with Texel (TXL) and French Berrichone du Cher (BDC) sheep breeds [5]. GWM is early maturing and has distinct meat and weather hardiness characteristics [3, 5, 6]. It is meaty at the hindquarters and loin, and in Northern Germany, the animals are typically grazed along dikes where they compact the soil to prevent erosion. The breed is currently not endangered but its flock book numbers have been decreasing over the years. It is therefore listed as a monitoring population and in Schleswig-Holstein, many flock owners are “hobby breeders”. Arguably, some of these breeders may retire in the near future due to their age and this may compound the problem of decreasing number of animals in the flock book.

Small populations have a higher risk of inbreeding and loss of genetic diversity. The consequence of inbreeding depression, which manifests in reduced survival and fertility of offspring of related individuals is long established [7–9]. Genetic analysis offers a tool for studying population demography and genetic patterns that can be applied in breed conservation management. Both pedigree and genome analyses have been used to characterise genetic diversity in several species. While pedigree analysis has some limitations, genome-based assessment is gaining wider acceptance due to advances in the development of single nucleotide polymorphism (SNP) chip technology. Recent application of SNP data analysis revealed a suitability of the OvineSNP50 BeadChip to infer population structure and genome-wide diversity in Russian [10] and Ethiopian [11] sheep, and in some meat sheep breeds across Europe [12]. In the European breeds, runs of homozygosity (ROH) distribution patterns and abundance were used to quantify genetic diversity [12]. The authors reported chromosomal ROH coverage variation but did not deliberately discuss the partitioning of total ROH inbreeding coefficient (F_ROH_) into chromosomal inbreeding. Following McQuillan et al.’s [13] original concept, Curik et al. [14] suggested the calculation of chromosomal inbreeding as the total length of all ROH addressed to a chromosome expressed as a proportion of length of the related autosomal chromosome covered by SNPs in chip. In estimating the effect of intrachromosomal inbreeding depression on female fertility, Martikainen et al. [15] made use of chromosomal inbreeding calculated in relation to the number (rather than length) of SNPs in ROH. Such a difference provides avenue for investigating the outcome of using different computational measures.

Given the historical background of GWM sheep, the objectives of the current study were to quantify genetic diversity in the breed by applying both pedigree and genomic information, and to investigate patterns of admixture between GWM and other breeds, especially those that breeders perceive as historically related to GWM. Furthermore, different measures of ROH-based inbreeding and patterns of chromosomal inbreeding variation in GWM were investigated.

## Materials and methods

### Data and data preparation

The GWM pedigree data consisted of 19,000 individuals (5,426 males and 13,574 females) born between 1970 and 2015 and were provided by the “Landeskontrollverband Schleswig-Holstein e.V.”. The data mainly covered animals from approved sheep breeding organisations in Schleswig-Holstein. The pedigree information comprised of individual and parent identification numbers, sex and birth date of individuals.

Blood samples were collected on 48 GWM individuals from across eight flocks for DNA extraction. Care was taken to possibly avoid the selection of closely related animals. Trained veterinarians performed the sample collection after a permission to carry out the genetic study had been granted by the Ministry of Energy Transition, Agriculture, Environment, Nature and Digitization in Schleswig-Holstein. Approval of the study was provided in consultation with the Animal Welfare Authority of the University of Kiel. From the blood samples, DNA was extracted following standard extraction protocol and all 48 individuals were genotyped with the OvineSNP50 BeadChip, which features 54,241 evenly spaced SNPs. Out of the 48 genotyped individuals, 31 had pedigree records and were born between 2009 and 2015. After the removal of all non-autosomal and non-annotated SNPs from the genotype dataset, PLINK [16] was used to filter out both individuals and SNPs with call rate below 90%. SNPs with minor allele frequencies lower than 5% or that deviate from Hardy Weinberg expectation (*P* value ≤ 0.0001) were excluded. Consequently, 46 individuals remained after filtering, all having the same 40,753 SNPs (hereafter, referred to as Data1) with average distance of 65.875 kb (minimum, 0.065 kb; maximum, 997.005 kb) and a mean r^2^ value of 0.209 between adjacent SNPs (S1 Table).

Publicly available genotype data at the “Web-Interfaced next generation Database dedicated to genetic Diversity Exploration” (WIDDE) [17] were obtained for the investigation of admixture between GWM and other breeds. These data covered 209 individuals belonging to nine breeds including BDC, German Texel (GTX), Border Leicester (BRL), New Zealand Romney (ROM), Suffolk (SUF), East Friesian White (EFW), Mouton Charolais (CHL), Spanish Merino-Estremadura (SME) and Spanish Merino-Andalusian (SMA) sheep (Table 1). The obtained data were merged with Data1 and further pruned for linkage disequilibrium (LD) applying the “—indep 50 5 2” PLINK [16] command. The resulting dataset, hereafter, referred to as Data2 consisted of 16,852 SNPs on 255 individuals.

**Table 1:** Description and source of publicly available genotype data on 209 individuals across nine sheep breeds.

Breeds	Abbreviation	Number	Data reference
Berrichone du Cher	BDC	19	[18]
German Texel	GTX	46	[19]
Border Leicester	BRL	48	[19]
New Zealand Romney	ROM	24	[19]
Spanish Merino (Andalusia)	SMA	7	[20]
Spanish Merino (Estremadura)	SME	13	[20]
Suffolk	SUF	19	[18]
East Friesian White	EFW	9	[19]
Mouton Charollais	CHL	24	[18]

### Pedigree analysis

To characterise the GWM population in terms of demography and possible unbalanced genetic contribution, five different population parameters including *Generation interval (L)*, *Inbreeding coefficient (F)*, *Effective population size (N*_*e*_*)*, *Effective number of founders* (*f*_*e*_) and *Effective number of ancestors*)(*f*_*a*_) were computed using ENDOG v4.8 [21]. A reference subpopulation consisting of animals born between 2012 and 2015 was defined for the most current generation in the pedigree. The quality of pedigree information was assessed by computing the pedigree completeness index following MacCluer et al. [22] and by calculating the complete generation equivalent (CGE) [21]. *L* refers to the average age of parents at the birth of the progeny that is kept for reproduction. This was calculated separately for each of the four gametic pathways namely sire to son (L_ss_), sire to daughter (L_sd_), dam to son (L_ds_) and dam to daughter (L_dd_). *F* is the probability of an individual having two identical alleles by descent and was calculated following Meuwissen and Luo [23]. To calculated *N*_*e*_, the individual inbreeding coefficients were regressed over the respective CGE and the resulting regression coefficient was considered as the rate of inbreeding (Δ*F*) [21]. Furthermore, *N*_*e*_ was obtained using the expression $N_{e}=\frac{1}{2\Delta F}$. When calculating *N*_*e*_ for the reference subpopulation, Δ*F* was approximated as $\Delta F=\frac{b}{1-(F_{t}-b)}$, where *F*_*t*_ is the average *F* of the given subpopulation and *b* is the regression coefficient as previously defined [21]. The *f*_*e*_ defines the number of equally contributing founders that would be expected to produce the same genetic diversity as in the population under study [24]. It can be calculated as:

$$f_{e}=1/\sum_{k=1}^{f}q_{k}^{2},$$

where *q*_*k*_ is the probability that a gene randomly sampled in the population originates from founder *k*, and *f* is the total number of founders [25]. The *f*_*a*_ refers to the minimum number of ancestors, not necessarily founders, explaining the complete genetic diversity of a population [25] and can be computed as follows:

$$f_{a}=1/\sum_{j=1}^{a}q_{j}^{2},$$

where *q*_*j*_ represents the marginal genetic contribution of ancestor *j*, i.e., the genetic contribution made by an ancestor that is not explained by previously chosen ancestors, and *α* is the total number of ancestors considered.

### Genomic analysis

#### Demography and population structure

For Data1, observed (H_o_) and expected (H_e_) heterozygosity values were calculated using the “—het” function in PLINK [16] and recent past effective population size estimates were calculated from the relationship between linkage disequilibrium (LD) and recombination rate using SNeP [26]. SNeP calculates *N*_*e*_ following Corbin *et al*. [27]:

$$N_{T(t)}={\left(4f(c_{t})\right)}^{-1}(E{\left[r_{adj}^{2}|c_{t}\right]}^{-1}-\propto ),$$

where *N*_*T*(*t*)_ is the effective population size *t* generations ago, calculated as *t* = (2*f*(*c*_*t*_))^−1^ [28], *c*_*t*_ is the recombination rate for a specific physical distance between SNPs estimated following Sved and Feldman [29], $r_{adj}^{2}$ is the LD value adjusted for sample size and α is a correction for the occurrence of mutations. The maximum distance between SNPs was set to 10 Mb to allow the estimation of *Ne* related to 5 generations back (*Ne*_*5Gen*_), which is considered the most recent [10, 30].

The clustering of Data1 samples with respect to flock or breeders was investigated using NetView [31, 32] analysis of pairwise genetic distances computed using PLINK [16]. NetView is a high-resolution network visualisation tool that detects fine-scale population structures based on genetic distances, and it requires a specification of the maximum number of nearest neighbours (K-NN) an individual can have. K-NN values of 10 and 50 were used to study fine- and large-scale genetic structures, respectively. Multidimensional scaling (MDS) and Netview analyses were performed on Data2 to investigate relatedness of GWM to the other breeds. To the same end, a model-based admixture analysis [33] was performed to determine the number of clusters and to assign individuals to these clusters. A 20-fold cross-validation procedure was performed for a range of k between 1 and 30 using the “cv-flag” of ADMIXTURE, and the k with the lowest cross-validation error was considered as the optimal number of clusters for Data2. Cluster assignments for k values ranging from 2 to 11 were graphically presented using Pophelper [34].

#### ROH inbreeding

ROH were detected in GWM (Data1) by applying a sliding window of 50 SNPs, allowing one possible heterozygous genotype, up to two missing genotypes, a minimum SNP density of 1 SNP every 100 kb and a maximum gap of 1 Mb between consecutive homozygous SNPs. The detection of ROH was performed considering the entire autosome and for each chromosome separately using PLINK [16]. After detection, total and chromosomal inbreeding coefficients were calculated for each individual following McQuillan et al.’s [13] original concept. Total individual ROH inbreeding coefficient was calculated in two ways, thus, in terms of ROH length (***F*_*ROH*_*L*_**) and based on the number of SNPs in ROH (***F*_*ROH*_*N*_**) as:

$$F_{ROH\_L}=\frac{\sum L_{ROH}}{L_{AUTO}}$$

$$F_{ROH\_N}=\frac{ROH\_SNP}{N\_SNP},$$

where ∑*L*_*ROH*_ refers to the total length of all ROH in the genome of an individual, *L*_*AUTO*_ is the length of the autosomal genome coverage by SNPs on the chip (2,644,827 kb), *ROH*_*SNP* is the number of SNPs in all detected ROH in an individual and *N*_*SNP* is the total number of SNPs on the autosomal genome under investigation. Chromosomal inbreeding was calculated in terms of ROH length either by dividing the sum of ROH on a given chromosome (∑*L*_*ROH*_*K*_) by the length of the respective chromosome (*L*_*AUTO*_*K*_) or by dividing ∑*L*_*ROH*_*K*_ by the length of autosomal genome (*L*_*AUTO*_) as:

$$F_{ROH\_KK}=\frac{\sum L_{ROH\_K}}{L_{AUTO\_K}}$$

$$F_{ROH\_KA}=\frac{\sum L_{ROH\_K}}{L_{AUTO}},$$

where *F*_*ROH*_*KK*_ and *F*_*ROH*_*KA*_ are chromosomal inbreeding coefficients relative to chromosome length and to autosome length, respectively. For each individual, the sum and average of *F*_*ROH*_*KA*_ and *F*_*ROH*_*KK*_, respectively, over all chromosomes were computed to mimic an individual’s total inbreeding coefficient. The summation of *F*_*ROH_KA*_ over all chromosomes is mathematically identical to *F*_*ROH_L*_, hence, *F*_*ROH_KA*_ was introduced to obtain exact estimates for the ranking of chromosomes based on inbreeding load. Finally, inbreeding estimates derived for the different scenarios were compared using linear regression and Pearson’s correlation (*r*_*p*_). The genome-based estimates were also compared with pedigree inbreeding (F_PED_) estimates. Based on *F*_*ROH_L*_ estimates, a second genomic effective population size (*N*_*e_ROH*_) was calculated in the same way as pedigree *N*_*e*_; by first regressing individual *F*_*ROH_L*_ estimates on CGE to calculate Δ*F*. Here, the absolute value of the regression coefficient was used to avoid a negative value of effective size.

## Results

### Pedigree based parameters

**Fig 1:**
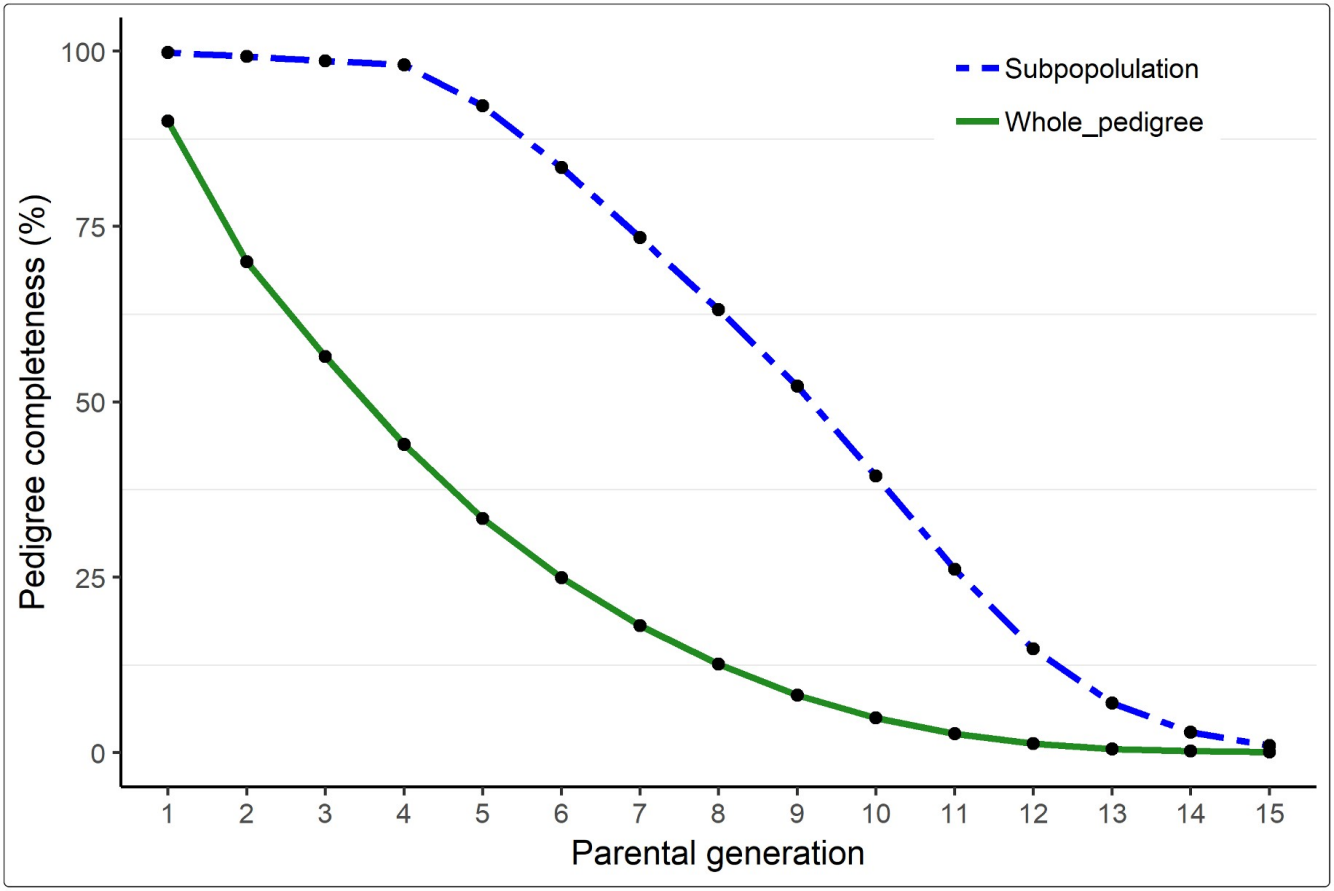
Pedigree completeness per parental generation computed for the entire German White-headed mutton pedigree (green) and for the reference subpopulation (blue). Parental generation 1 represents parents, 2 represents grandparents, etc. Animals were born between 1970 and 2015.

Pedigree completeness decreased with increasing pedigree depth (Fig 1). The quality of pedigree information was higher in the reference subpopulation than in the entire pedigree data. For instance, at the 5^th^ parental generation, pedigree completeness was 92.2% in the reference subpopulation and 33.4% in the entire pedigree.

Estimates of mean CGE were 3.67 (0.0–10.87) for the entire GWM pedigree, 8.52 (2.71–10.87) for the reference subpopulation and 8.05 (6.43–10.87) for the 31 genotyped individuals with pedigree information. Average *L* calculated based on 9,310 animals was 3.24 ± 2.13 years (Table 2). Generation interval was generally shorter in sires than in dams.

**Table 2:** Number of animals, generation interval and standard deviation (SD) for the four gametic pathways of the German White-headed Mutton sheep population.

Gametic Pathway	Number of animals	Interval ± SD (years)
Sire to son (L_ss_)	576	2.66 ± 1.39
Sire to daughter (L_sd_)	3046	2.62 ± 1.40
Dam to son (L_ds_)	660	4.16 ± 2.54
Dam to daughter (L_dd_)	5028	3.56 ± 2.37
**Average *L***	**9310**	**3.24 ± 2.13**

Table 3 presents estimates of pedigree-based inbreeding parameters. Average *F* was highest in the reference subpopulation (3.50%) and the corresponding *N*_*e*_ was smaller (99). The average *F* for the whole population was 1.02% and low, however, only 7,356 individual had *F* values greater than zero. Considering only inbred animals, average *F* was 2.62%. For the 31 genotyped individuals with pedigree information, average *F* was 2.94% (not in Table). In both the whole population and reference subpopulation, *f*_*e*_ was larger than *f*_*a*_.

**Table 3:** Parameters describing inbreeding, effective size and the concentration of genes in the whole and subpopulations of the German White-headed Mutton sheep pedigree.

Parameter	Whole population	Subpopulation
Number of reference animals	17,078	849
Mean inbreeding coefficient (*F*) (%)	1.02	3.50
Effective population size (*N*_*e*_)	132	99
Effective number of founders (***f*_*e*_**)	186	87
Effective number of ancestors (***f*_*α*_**)	144	31
*f*_*a*_ / *f*_*e*_	0.77	0.36

### Genome-based assessment

#### ROH inbreeding estimates and distribution

The total number of ROHs detected considering all individual was 381. The number of ROH per individual ranged from 3 to 26 with an average of 8.28. Average length of ROH and number of SNPs in ROH were 14,506.67 kb (5,103.62 kb—68,228.71 kb) and 228.52 (100–1055), respectively. The correlation between the length of ROH and number of SNPs in ROH was 0.99 (P-value < 0.001).

Descriptive statistics of ROH inbreeding estimates for the different measures are given in Table 4. Inbreeding estimates calculated for *F*_*ROH_L*_ ranged from 1.07% to 20.19% with an average of 4.54% for the 46 individuals. The *F*_*ROH_L*_ estimates were slightly lower than those calculated based on the number of SNPs in ROH. Slightly lower average inbreeding estimates being 4.26% and 3.76% for n = 46 and n = 31, respectively, were found for chromosomally derived inbreeding calculated relative to chromosome length. The *F*_*ROH_KK*_ estimates were averaged over chromosomes to arrive at an individual’s total ROH inbreeding coefficient.

**Table 4:** Inbreeding coefficients estimated from the runs of homozygosity (ROH) applying different computational measures on the German White-headed Mutton individuals.

Parameter	Mean (min—max) %	Mean (min—max) %
	(n = 46)	(n* = 31)
**F_ROH_L_**	4.54 (1.07–20.19)	3.89 (1.07–13.24)
**F_ROH_N_**	4.64 (1.12–20.94)	3.95 (1.12–13.30)
**∅F_ROH_KK_**	4.26 (0.44–15.78)	3.76 (0.44–14.53)

*: only individuals with pedigree information.

Ø: average over all autosomes per individual.

**Fig 2:**
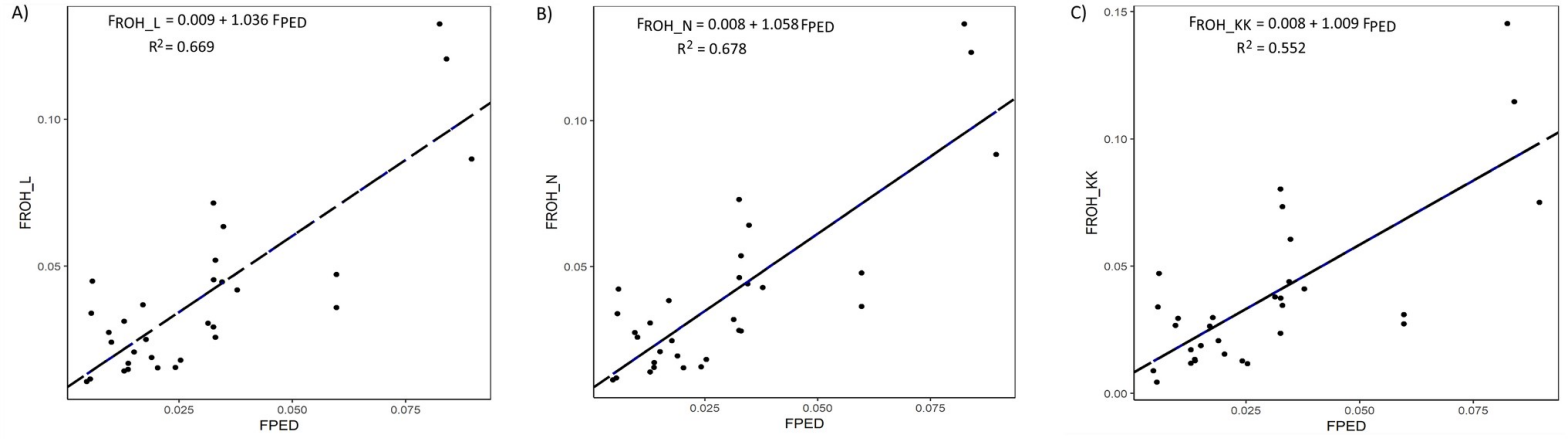
Regression plot of A) F_ROH_L_ on F_PED_, (B) F_ROH_N_ on F_PED_, and (C) F_ROH_KK_ on F_PED_ for 31 German White-headed mutton individuals.

For the different ROH computational measures, extremely high correlation coefficient estimates (above 0.96) were found for all comparisons (Table 5), and these methods effectively predicted F_PED_ (Fig 2). The lowest correlation (*r*_*p*_ = 0.74) was found between F_PED_ and *F*_*ROH_KK*_.

**Table 5:** Correlation coefficients between different measures of inbreeding calculated based on 31 German White-headed Mutton individuals (P-values < 0.001 in all cases).

	**F_PED_**	**F_ROH_L_**	**F_ROH_N_**	**F_ROH_KK_**
**F_PED_**	1.0000	0.8179	0.8235	0.7428
**F_ROH_L_**		1.0000	0.9995	0.9674
**F_ROH_N_**			1.0000	0.9678
**F_ROH_KK_**				1.0000

Based on the mean inbreeding estimates, *F*_*ROH_KA*_ and *F*_*ROH_KK*_ ranked chromosomes differently (S2 Table). Chromosomes 1, 2 and 3 had the highest mean inbreeding under *F*_*ROH_KA*_ while for *F*_*ROH_KK*_, the highest estimates were found for chromosomes 9, 20 and 7.

#### Population structure

**Fig 3:**
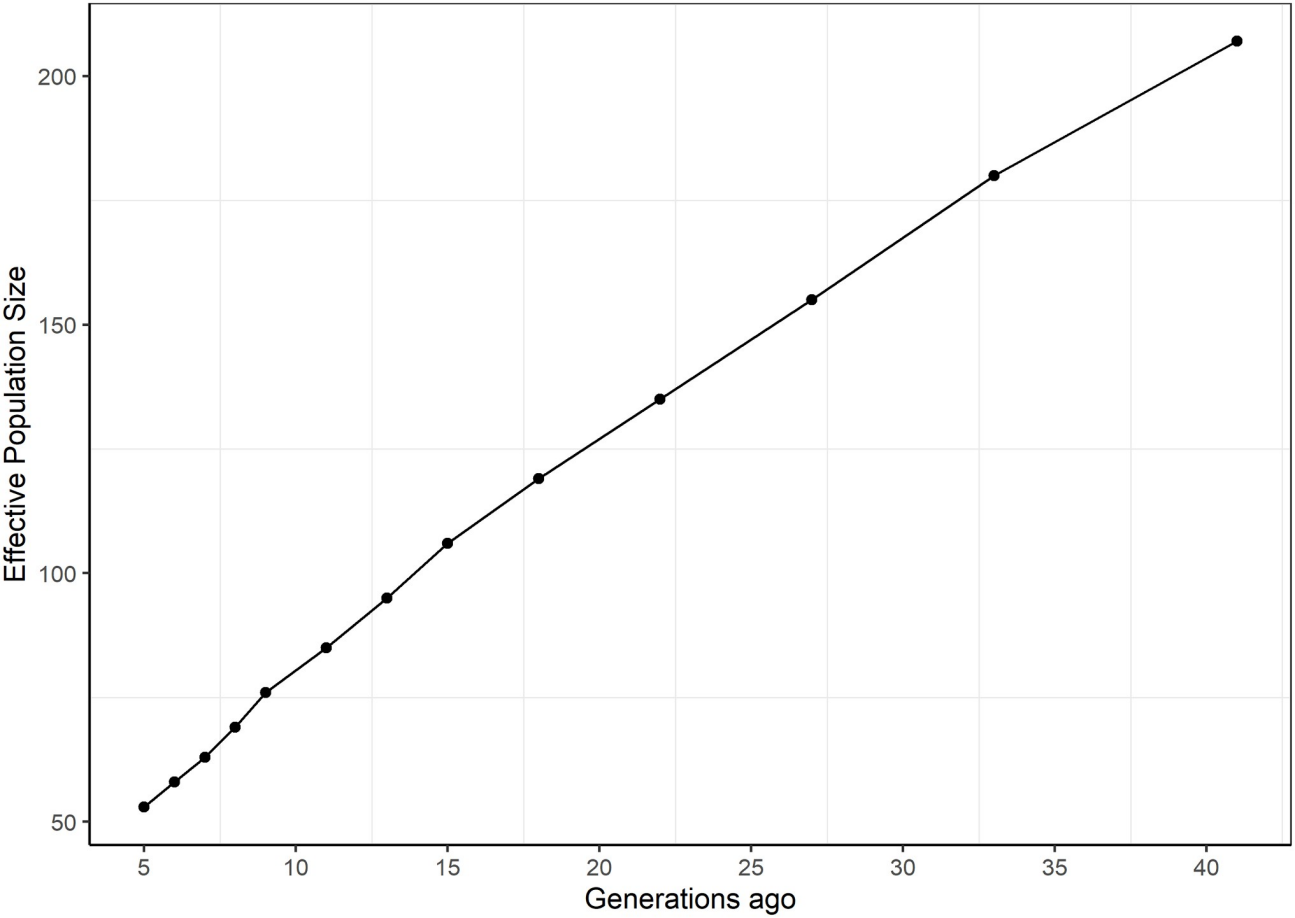
Trends in LD based effective population size across 41 generations of the German White-headed Mutton population.

The H_o_ estimate was on overage 0.387 (0.323 to 0.411) compared to a value of 0.376 for H_e_. The estimate of effective population size was 50.4 for *N*_*e_ROH*_. For the LD-based method, *N*_*e*_ was high in distant generations, about 207 at 41 generations ago (*N*_*e41Gen*_) but declined to 53 at 5 generations ago (Fig 3).

**Fig 4:**
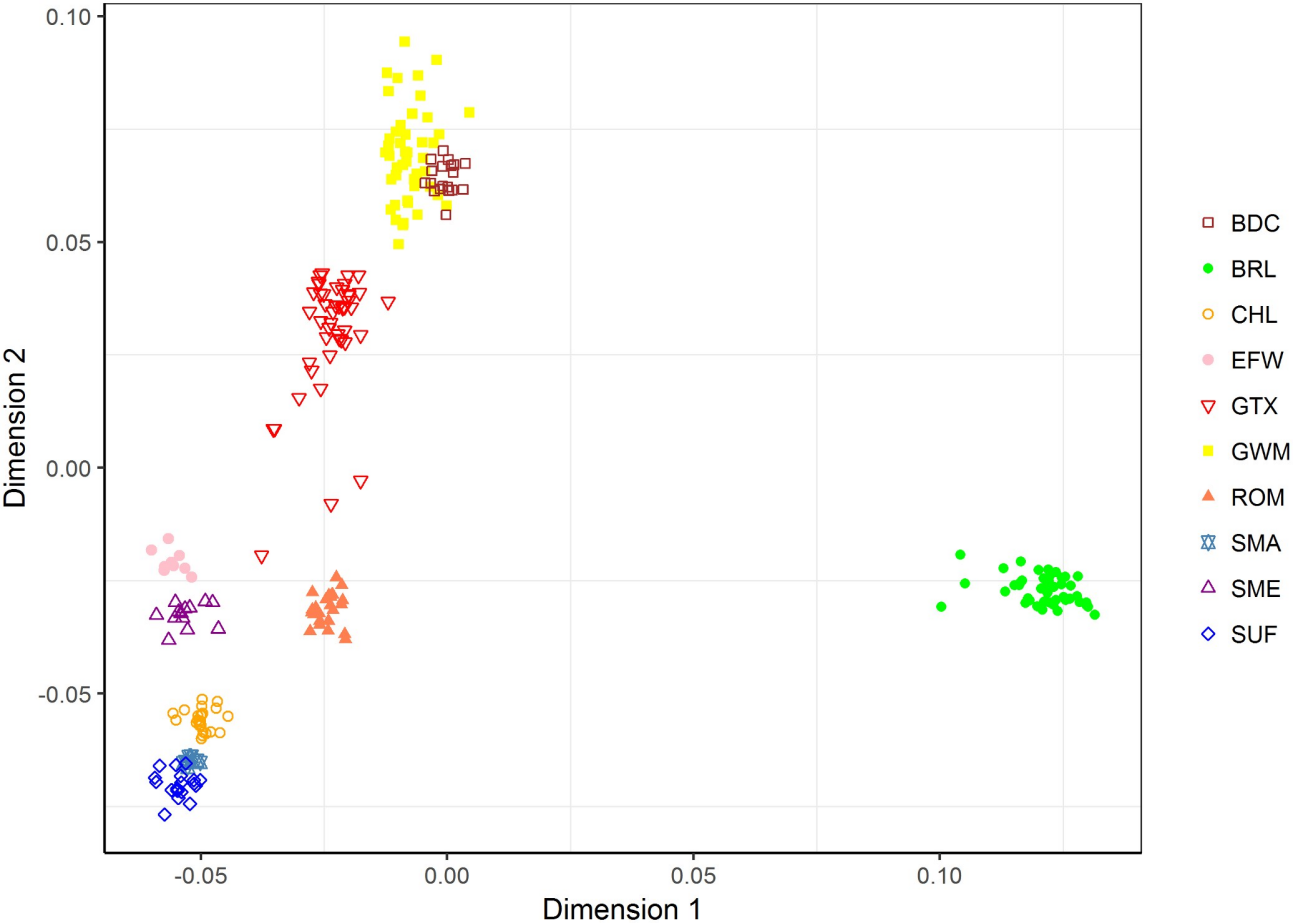
Multidimensional scaling plot of pairwise IBS distances among samples of 10 sheep breeds analysed based on 16,852 SNP markers (Data2). Breeds are distinguished by colour and shape.

The between-breed MDS analysis involving all 10 breeds revealed a partial clustering together of GWM and BDC samples as shown in Fig 4. The joint cluster has a closer proximity to GTX samples than to samples of all other breeds. The English breed (BRL) was distantly separated on dimension 1.

**Fig 5:**
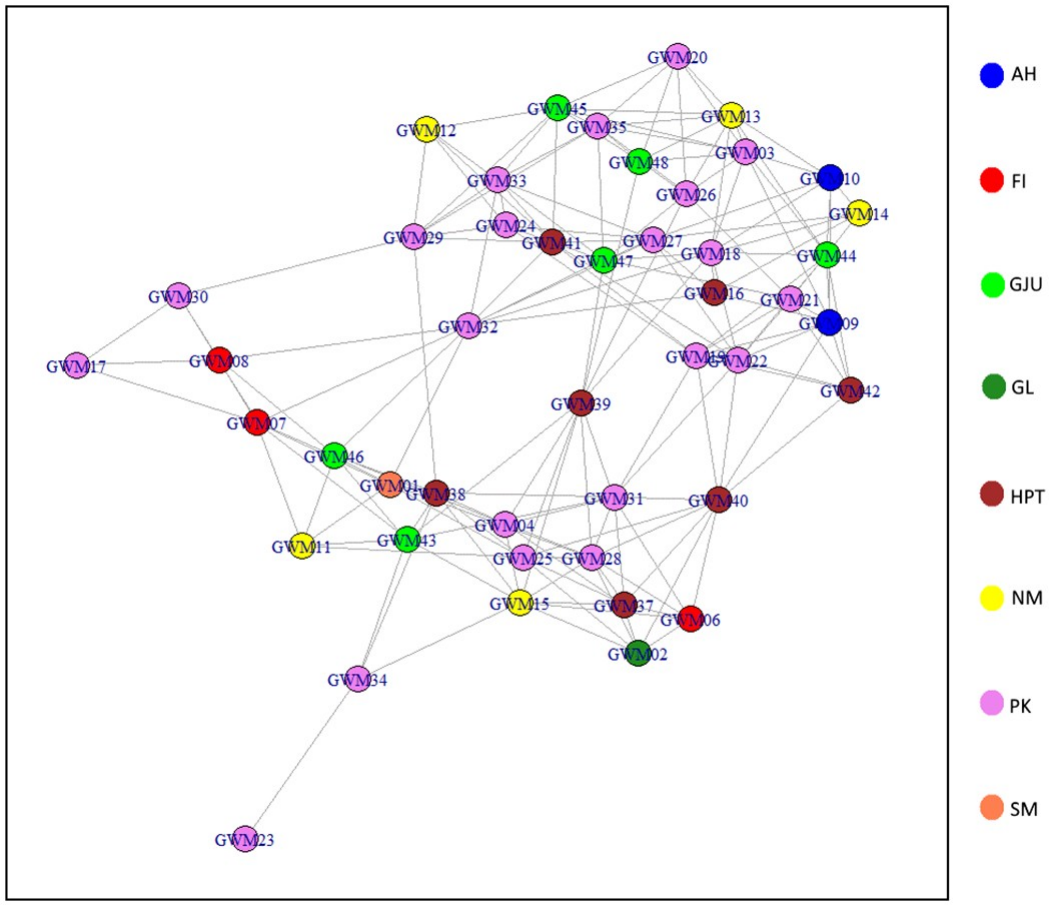
Network visualisation of the connectedness between German White-headed Mutton (GWM) individuals sampled from across eight breeders. Colours represent breeders/flocks.

Network visualisation of GWM genotypes did not show a clear separation by breeders, rather an interconnectivity of network among samples from different breeders as shown in Fig 5.

**Fig 6:**
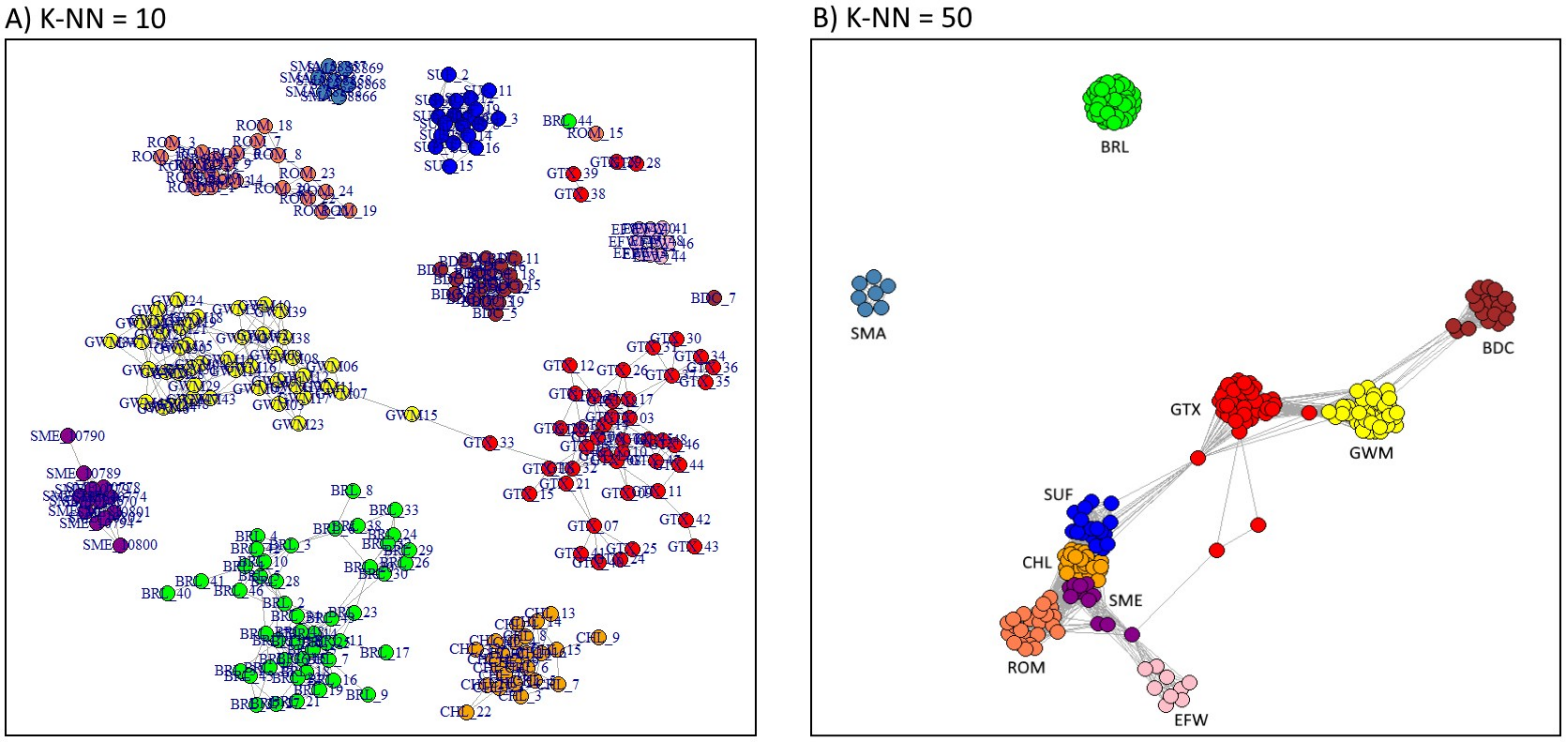
Fine- and large-scale network visualisation of relatedness in the 10 sheep breeds.

Pooling all 10 breeds together in the NetView analysis, well-defined clusters comparable to those in the MDS plot of Data2 were formed (Fig 6). For K-NN = 10, a network connection was found between a GWM individual (ID = GWM15) and an individual belonging to the GTX breed (ID = GTX_33). Few samples including 4 GTX, 1 BRL, 1 ROM and 1 BDC were placed outside their groups probably due to high level of dissimilarity. An increase of K-NN to 50 resulted in a rapid clustering of samples within breed and the condensation of populations as revealed in the massive network connectivity between breeds. GWM samples formed a cluster highly connected to, and sandwiched between GTX and BDC. On the other hand, BRL and SMA clusters were distinctly separated from the rest of the breeds.

**Fig 7:**
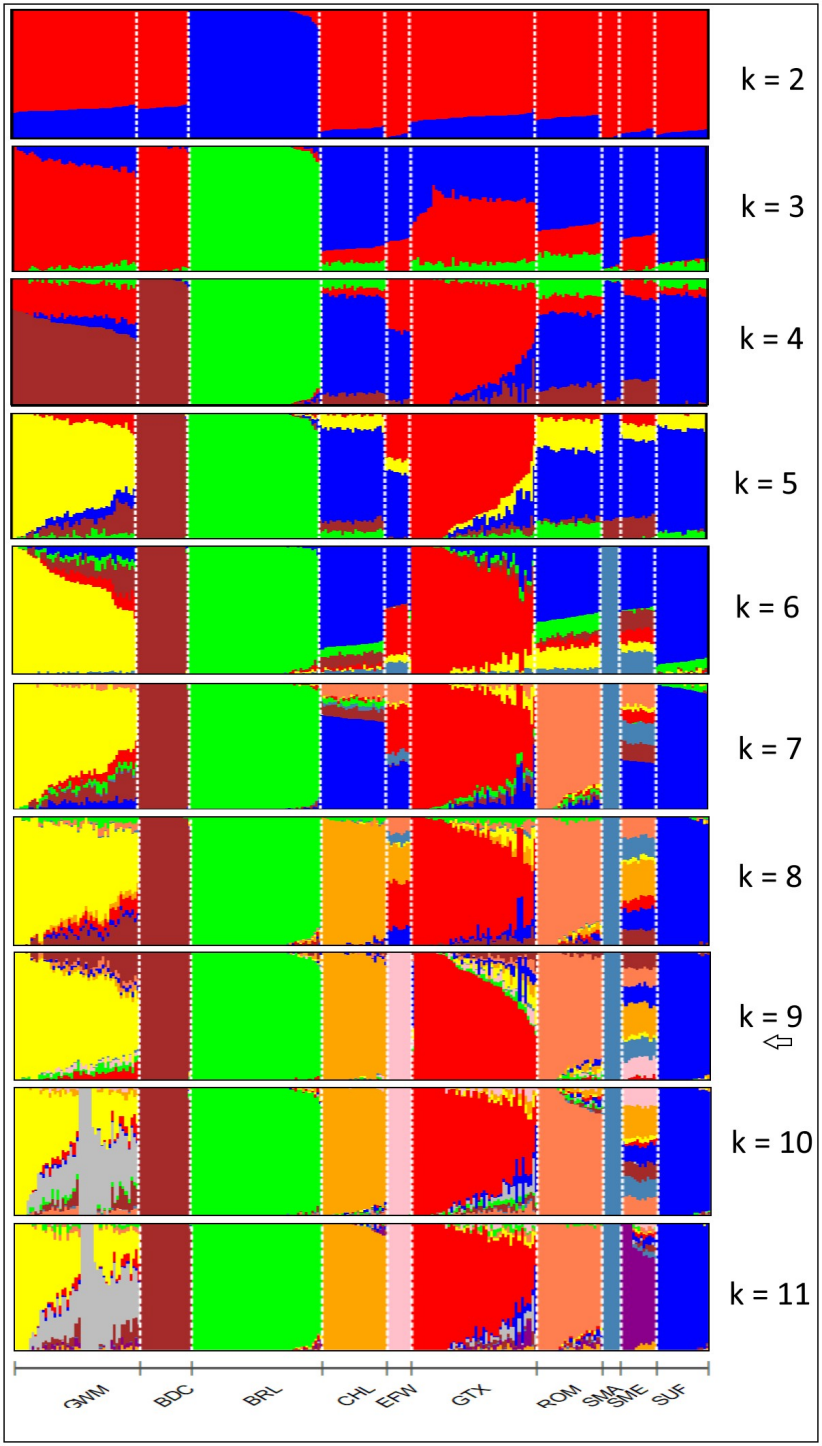
Admixture analysis of 10 sheep breeds. Results are presented for k = 2–11 and the optimal number of clusters (k = 9) is indicated with an arrow.

**Fig 8:**
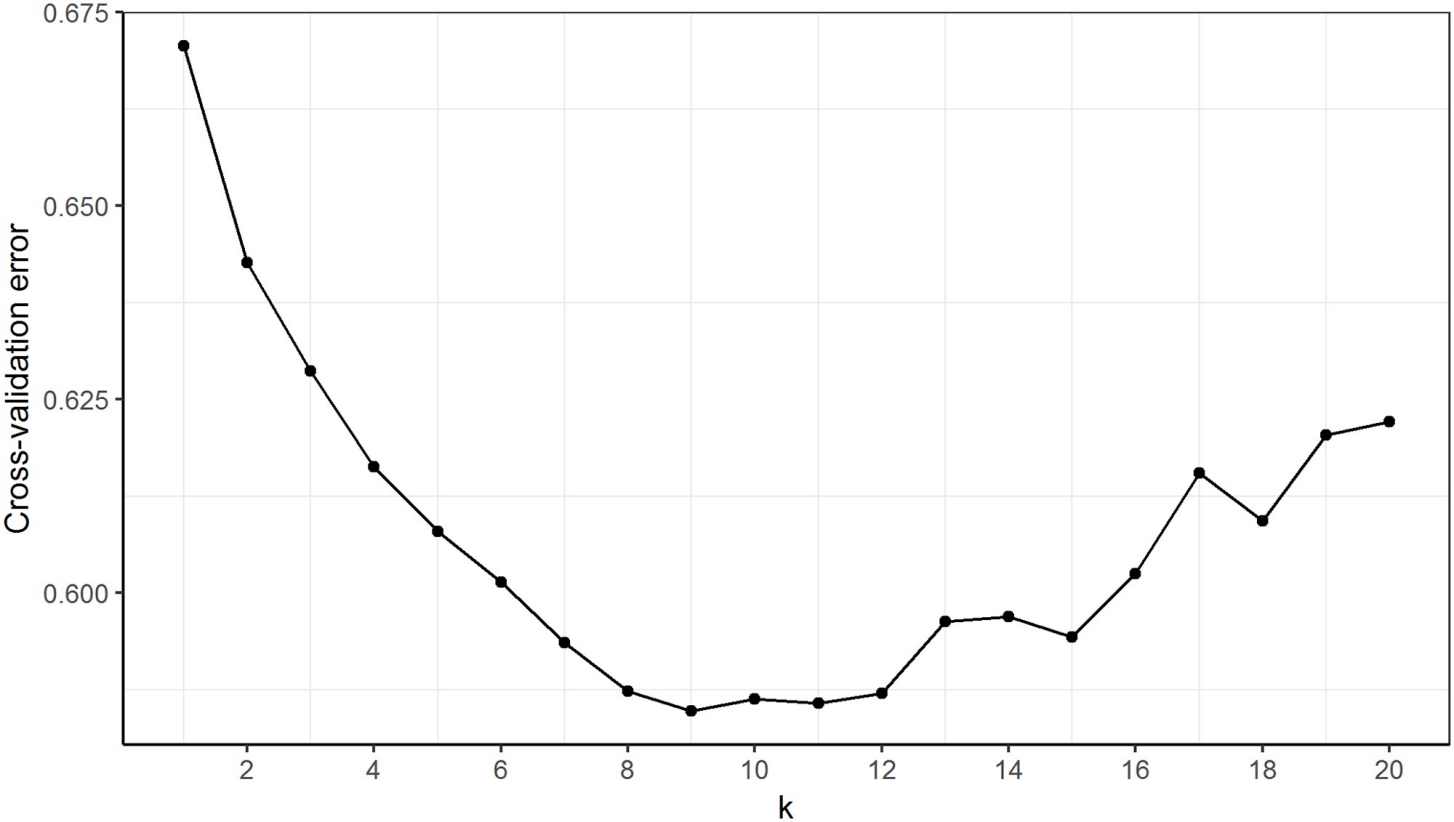
Development of the cross-validation for different number of clusters (k) with k = 9 showing the lowest error estimate.

Cluster assignment (Fig 7) at k = 2 of the admixture analyses was consistent with the findings of dimension 1 in the MDS plot for Data2, where BRL was distinctively separated from the rest of the breeds. At k = 3, GWM and BDC tended to share a high degree of genetic background (red) which was also present in the amount of about 50% in GTX. At this cluster level, uniformity of genetic background within breed was high in BDC, BRL, SMA and SUF. Further increase in k allowed for the visualisation of uniqueness of other breeds with uniform genetic background and those that are admixed. For instance, uniqueness was achieved at k = 7 for ROM, k = 8 for CHL, k = 9 for EFW and k = 11 for SME. Overall, GWM and GTX were the most admixed breeds. In the cross-validation procedure, k = 9 showed the lowest validation error as shown in Fig 8. Therefore, nine populations are considered most probable for Data2. At k = 9, GWM (predominantly yellow) showed high proportions of ancestry from BDC (brown) and to a lesser extent, genetic fractions from GTX (red), and BRL (green). Specks of genetic fractions from the SUF and EFW were also evident in GWM.

## Discussion

The present study sought to shed light on the genetic diversity and breed composition of GWM, and furthermore, compare different measures of computing inbreeding coefficients from ROH. The different ROH measures showed similar characteristics and were able to predict pedigree inbreeding with somewhat variable accuracies. A relatively high level of genetic diversity and some degree of gene flow events in GWM are hereby discussed.

### Different ROH inbreeding approaches

ROH-based inbreeding coefficients have been widely calculated in relation to the proportion of length of autosomal genome in ROH, given predefined ROH detection criteria [10, 12, 35–38]. The use of the number of SNPs in ROH rather than the length of ROH as was the case in Martikainen et al. [15] raises questions as to whether these two different approaches provide identical results. For the 381 detected ROH across all genotyped individuals in this study, a quick test using Pearson’s correlation coefficient indicated a near perfect relationship between the two variables: length of ROH vs. number of SNPs in ROH. This is not surprising, and can be explained as the consequence of the number of SNPs in each autosome being proportional to autosome length and SNPs being evenly spaced. The highly significant correlation coefficient between inbreeding estimates of F_ROH_L_ and F_ROH_N_ further substantiates the similarity of these measures. F_ROH_L_ and F_ROH_N_ predict pedigree inbreeding with about the same level of accuracy as shown in Fig 2. The strength of the correlation between F_PED_ and either F_ROH_L_ or F_ROH_N_ in this study was 0.82 and higher than what was reported by Purfield et al. [12] for different ROH length categories in six commercial sheep breeds where the estimated correlations fall in the interval between 0.12 and 0.76. The different depths of pedigree may have contributed to the different estimates. In the current study, the mean CGE of pedigree was 8.05 while the pedigree of Purified et al. [12] had a mean CGE of 6.5. Consistent with our findings, Burren et al. [35] reported a correlation coefficient of 0.81 for three Swiss goats breeds namely Chamois colored, Saanen and Toggenburg. These authors, however, reported lower correlations for other breeds especially, for those having pedigrees with completeness below 90%. All the genomic inbreeding estimators predicted inbreeding values of approximately 1% when pedigree inbreeding was zero as shown in Fig 2. This confirms the findings of previous studies [30, 39] in which F_PED_ was downgraded for its failure to capture autozygosity of ancient origin. Another advantage of using ROH is that total F_ROH_ can be conveniently partitioned into values for individual chromosomes or for specific chromosomal segments [40]. That way, a localised effect of inbreeding on a phenotypic trait can be investigated. For instance, Martikainen et al. [15] found the increase in F_ROH_ on chromosomes 2, 18 and 22 to be significantly associated with the intervals from first to last insemination in heifers. Notably, slight differences existed between the inbreeding coefficient estimates derived from *F*_*ROH_L*_ and *F*_*ROH_KK*_. Nevertheless, the observed differences were statistically not significant and besides, the correlation among these measures was high (*r*_*p*_ = 0.967). From a comparison of the mean estimates, *F*_*ROH_KK*_ tended to underestimate inbreeding, but it seems logical to calculate chromosomal inbreeding relative to the chromosome in question. A realised advantage of *F*_*ROH_KK*_ is its ability to capture the actual variability in chromosomal inbreeding estimates among individuals. If one is interested in chromosome specific variability rather than variation across the entire autosomal genome, *F*_*ROH_KK*_ can be considered.

### Genetic diversity and structure

The quality of pedigree information in terms of depth and completeness has a tremendous influence on the reliability of pedigree-based inbreeding estimates [25]. Pedigree completeness decreased with increasing depth in the current study. There was almost no information on ancestry at the 15^th^ parental generation, which is about 49 year ago. This corresponds to the year of birth of the oldest animals in the pedigree, which was 1970. GMW was formally recognised as a new breed in 1885 [4], however official pedigree recording and herdbook management probably started much later. The difference in mean CGE estimates between the whole pedigree and animals born in the most current generation being 3.67 vs. 8.52, respectively, is an indication of improved pedigree management in the breed in recent years.

Disparity between inbreeding estimates for the entire pedigree and for the reference subpopulation could have arisen from an increase of inbreeding in the most recent generation. In contrast, inbreeding in the whole pedigree may have been underestimated due to low pedigree quality. Consistently, the estimated *F* for only inbred animals was slightly higher. In Gute sheep, Rochus and Johansson [41] reported the reverse situation where inbreeding estimate for the entire pedigree was 3.8% and higher than an estimate of 1.8% for a selected subpopulation. In the previous study, however, the pedigree was more complete in the entire population, and the subpopulation was not necessarily composed of animals in the most recent generation. Our pedigree-based inbreeding estimate of 3.50% in the most recent generation was confirmed by the genomic estimate which was 3.89% for 31 genotyped animals. Note that F_ROH_L_ is identical to the generally used F_ROH_ computational measure. From a genomic viewpoint, the observed autozygosity in GWM is generally lower than previous findings for six commercial meat sheep breeds in which the three most homozygous individual in the sample population namely Suffolk, Belclare and Texel had, on average, 31.5% of their autosome covered in ROH [12]. The testing and exchange of superior rams or sons thereof might be a major reason for a lower inbreeding level in GWM.

As expected, about 33% of total inbreeding can be mapped to chromosomes 1, 2 and 3, which had genome coverage of 11.33%, 9.95% and 9.17%, respectively as depicted in S2 Table. However, it could be that the largest chromosomes harbour most genes that have been favoured by selection in the breed, but confirming this requires other investigations that go beyond the scope of this study. Nevertheless, other studies have investigated the presence of selective signatures on the ovine genome [12, 18, 19]. High number of ROH were frequently found overlapping selection regions [12]. Additionally, the authors identified several selection signatures in regions, especially on chromosome 2, harbouring genes some of which enhance muscling and weight gain. Conversely, on chromosome 10 which ranked 17^th^ in terms of inbreeding burden, a comprehensive study of 74 world-wide breeds detected the highest selection signal in a region close to a relaxin family-like peptide receptor 2 gene associated with the absence of horn in sheep [19]. It is needful to consider the different breed characteristics in the different studies when making comparisons.

The observed low level of inbreeding in GWM is consistent with the high level of genetic diversity in the breed. Our H_e_ estimate of 0.376 is higher than previously reported estimates for other German sheep breeds which were 0.33 and 0.35 for Black-headed Mutton and Merinolandschaf, respectively, and for many other worldwide breeds [19, 20]. The mixed genetic background of GWM may play a role in the high genetic diversity observed. The pedigree-based estimated effective population size did not show any sign of GWM being “at risk”. In both the entire and most current populations, *N*_*e*_ was larger than the threshold value of 50. Below this critical *Ne*, the fitness of a population is threatened [42, 43]. Our estimated genome-based *Ne* values for most recent generations were at the lower boarder of the threshold value signalling the need for breed monitoring. Larger estimates of *N*_*e5Gen*_ ranging from 65 to 543 were found for several Russian sheep breeds [10] with different characteristics and population histories. The regression of individual *F*_*ROH*_ estimates on CGE to calculate the rate of inbreeding, which was subsequently plugged into the usual equation ($N_{e}=\frac{1}{2\Delta F}$) to determine *Ne* in the current study is not new. Hillestad et al. [44] calculated *Ne* in a similar manner, by first regressing the natural logarithm of (1-*F*_*ROH*_) on year of birth to produce inbreeding rates. They concluded that their ROH-based estimate of *Ne* for Norwegian Red cattle being 165, resembled the estimate previously made by the industry based on pedigree information which was 194. Rodríguez-Ramilo et al. [45] did a similar study on French dairy sheep breeds with similar results. Nevertheless, in the current study, the ROH-based *Ne* differed from the pedigree-based estimate but was very similar to the observed LD-based value. A small founding population could explain the small recent effective population size in GWM. Besides, the effective size at *N*_*e41Gen*_, which dates back to around the time of breed recognition [4], considering a constant *L* of 3.25 years was already small (Fig 3).

Within the GWM population, interconnectedness between genotypes of different flocks may imply a breeding management system where animals, especially rams are either exchanged or sold between breeders. The deliberate exchange of animals between different flocks in an organised mating scheme can be beneficial in restricting inbreeding rates. This was demonstrated in a study in which low inbreeding rates were found for rotational mating schemes in which rams were exchanged between flocks [46]. The current study revealed varying degrees of genetic similarity between the breed of interest and other sheep breeds. Given the historical background of GWM [4], BRL was included in the analysis to investigate the presence of English ancestry in the breed. The model-based admixture analysis provided evidence of English background in GWM, and although not in high proportion, this was found at all cluster levels. In the MDS and NetView analyses, however, the relationship between the two breeds was not detected. Furthermore, our results on all three measures of population structure revealed a comparatively high level of relationship between GWM, and BDC and GTX. This is consistent with literature in which BDC and Texel sheep were the last reported breeds used to further improve GWM [5]. BDC is known to perform well in carcass quality, hence a good choice as parental breed that also contributes about 6% to the Polish White-headed mutton breed [47]. The admixture results provided much insight into the extent of ancestor sharing among the breeds. Overall, the BDC fraction was highest in GWM indicating a more recent unidirectional gene flow event between the two breeds. The proportion of GTX background detected in GWM is comparatively small and this was expected since GTX is not the original TXL breed used in the genetic improvement programme. GWM also harbours specks of genetic fractions from other breeds including SUF, EFW and ROM. To our knowledge, ancestor sharing between GWM and these latter breeds are not well documented. The pattern of genetic background in GWM as revealed beyond k = 9 of the admixture analyses reemphasizes the breed’s crossbred nature and this is in conformity with the observed high level of genetic diversity.

## Conclusion

Our results showed a high level of genetic diversity accompanied by a considerably low inbreeding level from both genomic and pedigree viewpoints. That notwithstanding, the effective population size estimates from both ROH and LD suggest the need of a monitoring strategy that would consolidate the genetic diversity in GWM sheep. Periodic update of the diversity status of GWM from pedigree and genomic analyses may complement the census-based monitoring approach, which is already in place. Incentivising breeders to maintain a larger gene pool of active breeding animals can enhance genetic diversity in the GWM population. The study also shed light on the relatedness of GWM to other breeds, particularly confirming the presence of BDC, GTX and BRL blood in the current population of GWM. Furthermore, using the GWM dataset, the computation of ROH inbreeding coefficient considering the number of SNPs in ROH proved comparable to the conventional use of the length of ROH in Mb.

## Supporting information

S1 Table Description of the number of informative SNPs, length covered by SNPs, average, minimum and maximum distances and average r^2^ (linkage disequilibrium) between adjacent markers of the 26 autosomes (Chr.) for Data1. (DOCX)

S2 Table Ranking of chromosomes based on their mean inbreeding estimates calculated relative to either entire autosome (F_ROH_KA_: Left) or to length of chromosome (F_ROH_KK_: Right). Cumulative inbreeding values and percentages are provided for F_ROH_KA_.
(DOCX)

S1 Data (ZIP)
