# Supplementary material for: Genetic diversity and the application of runs of homozygosity-based methods for inbreeding estimation in German White-headed Mutton sheep
Source: PLoS One. 2021 May 6;16(5):e0250608. doi: 10.1371/journal.pone.0250608 (PMC8101715; doi:10.1371/journal.pone.0250608)
Supplement: S1 Table — (DOCX) [file pone.0250608.s001.docx]

**S1 Table. Description of the number of informative SNPs, length covered by SNPs, average, minimum and maximum distances and average r^2^ (linkage disequilibrium) between adjacent markers of the 26 autosomes (Chr.) for Data1**.

| Chr. | Number of SNPs | Length (kb) | Average distance (kb) | Minimum distance (kb) | | Maximum distance (kb) | Average r^2^ |
| --- | --- | --- | --- | --- | --- | --- | --- |
| 1 | 4564 | 299592.50 | 65656.91 | 5291 | 913190 | | 0.225 |
| 2 | 4294 | 263106.10 | 61052.61 | 1936 | 737756 | | 0.232 |
| 3 | 3893 | 242554.00 | 61993.91 | 5400 | 725916 | | 0.226 |
| 4 | 2146 | 127111.60 | 59259.47 | 5405 | 593064 | | 0.209 |
| 5 | 1830 | 115958.40 | 63399.88 | 65 | 890151 | | 0.229 |
| 6 | 1996 | 129012.80 | 62627.67 | 5342 | 871646 | | 0.234 |
| 7 | 1735 | 108670.60 | 62099.00 | 5303 | 978541 | | 0.233 |
| 8 | 1624 | 97781.11 | 60247.14 | 5385 | 964918 | | 0.213 |
| 9 | 1675 | 100630.90 | 60114.01 | 5298 | 789815 | | 0.224 |
| 10 | 1475 | 94097.18 | 59846.56 | 5286 | 997005 | | 0.214 |
| 11 | 893 | 66863.52 | 74959.11 | 5300 | 970421 | | 0.213 |
| 12 | 1332 | 86031.14 | 63775.02 | 5462 | 713377 | | 0.228 |
| 13 | 1286 | 88855.06 | 69147.91 | 5478 | 902996 | | 0.232 |
| 14 | 885 | 68749.33 | 76353.93 | 5283 | 873165 | | 0.194 |
| 15 | 1304 | 89836.08 | 67573.64 | 5414 | 996755 | | 0.196 |
| 16 | 1223 | 77077.93 | 63075.23 | 5908 | 499372 | | 0.189 |
| 17 | 1103 | 78441.81 | 71181.31 | 5413 | 562710 | | 0.204 |
| 18 | 1138 | 71850.31 | 63192.89 | 5366 | 675361 | | 0.182 |
| 19 | 951 | 64794.55 | 68204.79 | 5678 | 472834 | | 0.217 |
| 20 | 873 | 55536.56 | 62496.84 | 5499 | 651579 | | 0.200 |
| 21 | 694 | 55137.89 | 76178.98 | 5616 | 886763 | | 0.195 |
| 22 | 871 | 54914.22 | 60594.48 | 5312 | 905861 | | 0.206 |
| 23 | 872 | 66238.78 | 76049.11 | 5657 | 715163 | | 0.190 |
| 24 | 577 | 44235.13 | 76797.10 | 5514 | 696926 | | 0.189 |
| 25 | 784 | 47928.72 | 61211.64 | 5514 | 589556 | | 0.174 |
| 26 | 735 | 49820.90 | 65660.36 | 5396 | 357477 | | 0.196 |
| Total | **40753** | **2,644,827.00** | **65874.98** | **65** | **997005** | | **0.209** |
