# Supplementary material for: Genetic diversity and the application of runs of homozygosity-based methods for inbreeding estimation in German White-headed Mutton sheep
Source: PLoS One. 2021 May 6;16(5):e0250608. doi: 10.1371/journal.pone.0250608 (PMC8101715; doi:10.1371/journal.pone.0250608)
Supplement: S2 Table — Cumulative inbreeding values and percentages are provided for FROH_KA. (DOCX) [file pone.0250608.s002.docx]

**S2 Table. Ranking of chromosomes based on their mean inbreeding estimates calculated relative to either entire autosome (F_ROH_KA_: left) or to length of chromosome (F_ROH_KK_: right). Cumulative inbreeding values and percentages are provided for F_ROH_KA_.**

|  | **F_ROH_KA_** | | | | | **F_ROH_KK_** |  |
| --- | --- | --- | --- | --- | --- | --- | --- |
| Chr. | Rank | Mean F | Cumulative F | Cumulative (%) |  | Mean F | Rank |
| chr1 | 1 | 0.005500 | 0.005500 | 12.11 |  | 0.048555 | 10 |
| chr2 | 2 | 0.005459 | 0.010959 | 24.12 |  | 0.054872 | 8 |
| chr3 | 3 | 0.004119 | 0.015078 | 33.19 |  | 0.044913 | 12 |
| chr9 | 4 | 0.003182 | 0.018260 | 40.19 |  | 0.083636 | 1 |
| chr7 | 5 | 0.002763 | 0.021023 | 46.28 |  | 0.067251 | 3 |
| chr6 | 6 | 0.002228 | 0.023251 | 51.18 |  | 0.045685 | 11 |
| chr8 | 7 | 0.002115 | 0.025366 | 55.84 |  | 0.057197 | 5 |
| chr4 | 8 | 0.001933 | 0.027299 | 60.09 |  | 0.040219 | 14 |
| chr15 | 9 | 0.001892 | 0.029191 | 64.26 |  | 0.055713 | 7 |
| chr16 | 10 | 0.001717 | 0.030908 | 68.04 |  | 0.058913 | 4 |
| chr20 | 11 | 0.001650 | 0.032558 | 71.67 |  | 0.078580 | 2 |
| chr18 | 12 | 0.001526 | 0.034084 | 75.03 |  | 0.056160 | 6 |
| chr5 | 13 | 0.001414 | 0.035498 | 78.14 |  | 0.032249 | 19 |
| chr12 | 14 | 0.001384 | 0.036882 | 81.19 |  | 0.042545 | 13 |
| chr19 | 15 | 0.001317 | 0.038198 | 84.08 |  | 0.053741 | 9 |
| chr13 | 16 | 0.001270 | 0.039468 | 86.88 |  | 0.037796 | 15 |
| chr10 | 17 | 0.001089 | 0.040557 | 89.28 |  | 0.030608 | 20 |
| chr23 | 18 | 0.000903 | 0.041460 | 91.26 |  | 0.036038 | 18 |
| chr22 | 19 | 0.000784 | 0.042243 | 92.99 |  | 0.037748 | 16 |
| chr21 | 20 | 0.000769 | 0.043012 | 94.68 |  | 0.036876 | 17 |
| chr17 | 21 | 0.000556 | 0.043568 | 95.90 |  | 0.018745 | 22 |
| chr14 | 22 | 0.000462 | 0.044030 | 96.92 |  | 0.017758 | 23 |
| chr24 | 23 | 0.000430 | 0.044460 | 97.87 |  | 0.025726 | 21 |
| chr11 | 24 | 0.000396 | 0.044856 | 98.74 |  | 0.015671 | 25 |
| chr25 | 25 | 0.000292 | 0.045148 | 99.38 |  | 0.016110 | 24 |
| chr26 | 26 | 0.000281 | **0.045429** | **100.00** |  | 0.014932 | 26 |
